# Adult intracranial pial arteriovenous fistulas: a case report and literature review

**DOI:** 10.3389/fsurg.2026.1759994

**Published:** 2026-04-20

**Authors:** Hong Chen, Ying Xu, Zhongyue Liu, Yugang Jiang, Ming Wang

**Affiliations:** 1 Department of Neurosurgery, The Second Xiangya Hospital, Central South University, Changsha, Hunan, China; 2 Hunan Cerebrospinal Vascular Disease Diagnosis and Treatment Center, The Second Xiangya Hospital, Central South University, Changsha, Hunan, China

**Keywords:** endovascular treatment, hematoma, microsurgical resection, pial arteriovenous fistula, venous pouch

## Abstract

Intracranial pial arteriovenous fistula (PAVF) is a rare high-flow cerebrovascular lesion defined by a direct shunt between pial/cortical arteries and a single draining vein or venous pouch without a nidus, adult cases are particularly uncommon. A 56-year-old woman presented with sudden severe headache after yoga. CT/MRI revealed a right frontal intracerebral hemorrhage associated with a giant venous pouch, and angiography demonstrated a single-channel PAVF fed by an MCA M2 branch with venous drainage to the superior sagittal and sphenoparietal sinuses. The patient underwent craniotomy for hematoma evacuation, microsurgical fistula disconnection, and venous pouch resection under indocyanine green angiography and FLOW800 guidance. Postoperative CT confirmed complete hematoma removal, and follow-up DSA on day 4 showed total obliteration of the fistula with no residual abnormal drainage. Pathology revealed a vascular malformation with focal calcification. She was discharged neurologically intact and remained symptom-free without recurrence on CTA at 3 months. Adult PAVF is extremely rare but carries a high risk of hemorrhage; early angiographic diagnosis and definitive flow disconnection yield excellent outcomes.

## Introduction

Pial arteriovenous fistula (PAVF) is a rare intracranial vascular malformation characterized by a direct, high-flow shunt between one or a few pial/cortical arteries and a single cortical vein or venous pouch, usually accompanied by venous varix or venous aneurysm, and notably lacking a typical nidus (1). Therefore, PAVF differs from conventional brain arteriovenous malformations (AVMs) and dural arteriovenous fistulas (DAVFs) in morphology, hemodynamics, and therapeutic strategy. Clinically, PAVF most commonly presents with intracranial hemorrhage, seizures, or headache (2, 3). It accounts for only about 1.6%–4.8% of intracranial vascular malformations and shows a strong predilection for children, whereas adult cases are distinctly uncommon (3, 4). Because PAVF has a poor natural history, the clinical suspicion of pial AVF, followed by prompt appropriate treatment is important (5). Here, we report an adult patient with PAVF complicated by intracranial hemorrhage who achieved a favorable outcome after craniotomy for hematoma evacuation and excision of the PAVF. A review of the relevant literature is provided to improve understanding of this entity.

## Case report

A 56-year-old woman was admitted with a 1-week history of headache. She reported sudden onset of severe headache while practicing yoga one week earlier, without nausea, vomiting, or limb weakness. No medical evaluation or treatment was sought at that time. After resting at home for two days without relief, she visited a local hospital where head imaging suggested a frontal lobe hemorrhage, raising concern for possible tumor-related hemorrhage. She was referred to our institution for further management. On admission, neurological examination was unremarkable. She had no history of head trauma or intracranial surgery.

**Figure 1:**
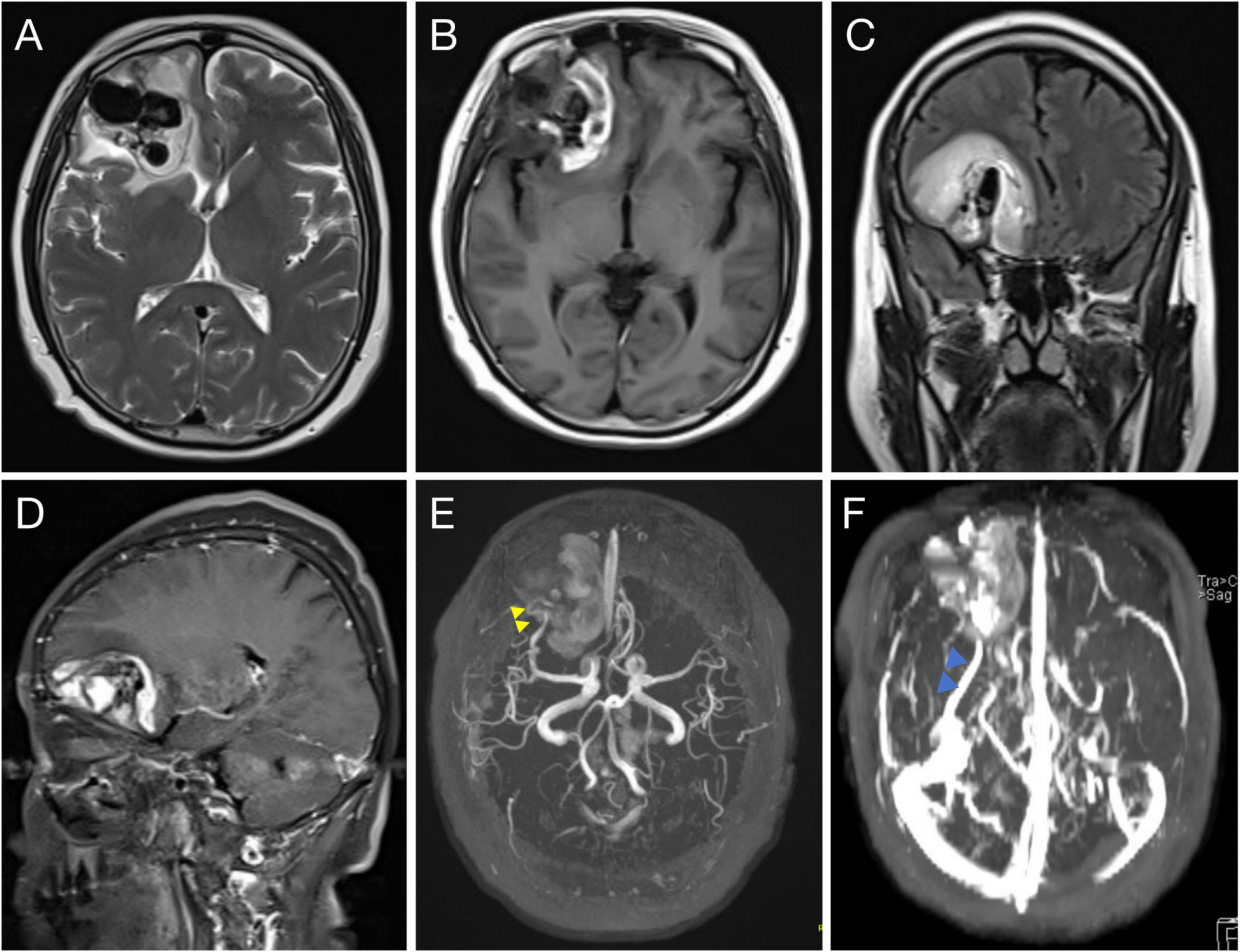
Preoperative T2-weighted MRI shows multiple prominent flow voids in the right frontal region **(A)**. T1-weighted MRI demonstrates right frontal intraparenchymal hemorrhage with heterogeneous signal within the hematoma **(B)** FLAIR shows marked perilesional edema **(C)**. Contrast-enhanced T1 MRI reveals heterogeneous enhancement **(D)**. MRA identifies an arterial branch from the right MCA M2 segment coursing through the lesion (yellow triangle) **(E)** MRV shows an enlarged draining vein (blue triangle) **(F)**.

Emergency brain MRI (Figures 1A,B) demonstrated an irregular mixed-signal mass in the right frontal lobe measuring approximately 46 mm × 39 mm. Patchy hyperintensity was noted within the lesion on T1-weighted images. Tortuous, round-like flow-void structures were seen within the lesion (Figure 1A). The T2-FLAIR sequence showed heterogeneous signal with surrounding patchy edema (Figure 1C). Contrast enhancement revealed irregular, prominent internal enhancement (Figure 1D). MR angiography demonstrated a small cortical branch arising from the right MCA M2 segment coursing through the lesion, with early venous drainage partly into the superior sagittal sinus (Figure 1E). MR venography showed that the draining vein to the sphenoparietal sinus was connected to the lesion (Figure 1F). The adjacent lateral ventricle was compressed, and the midline structures were mildly shifted to the left (Figures 1B,C). These findings suggested right frontal lobe hemorrhage with a possible vascular malformation.

**Figure 2:**
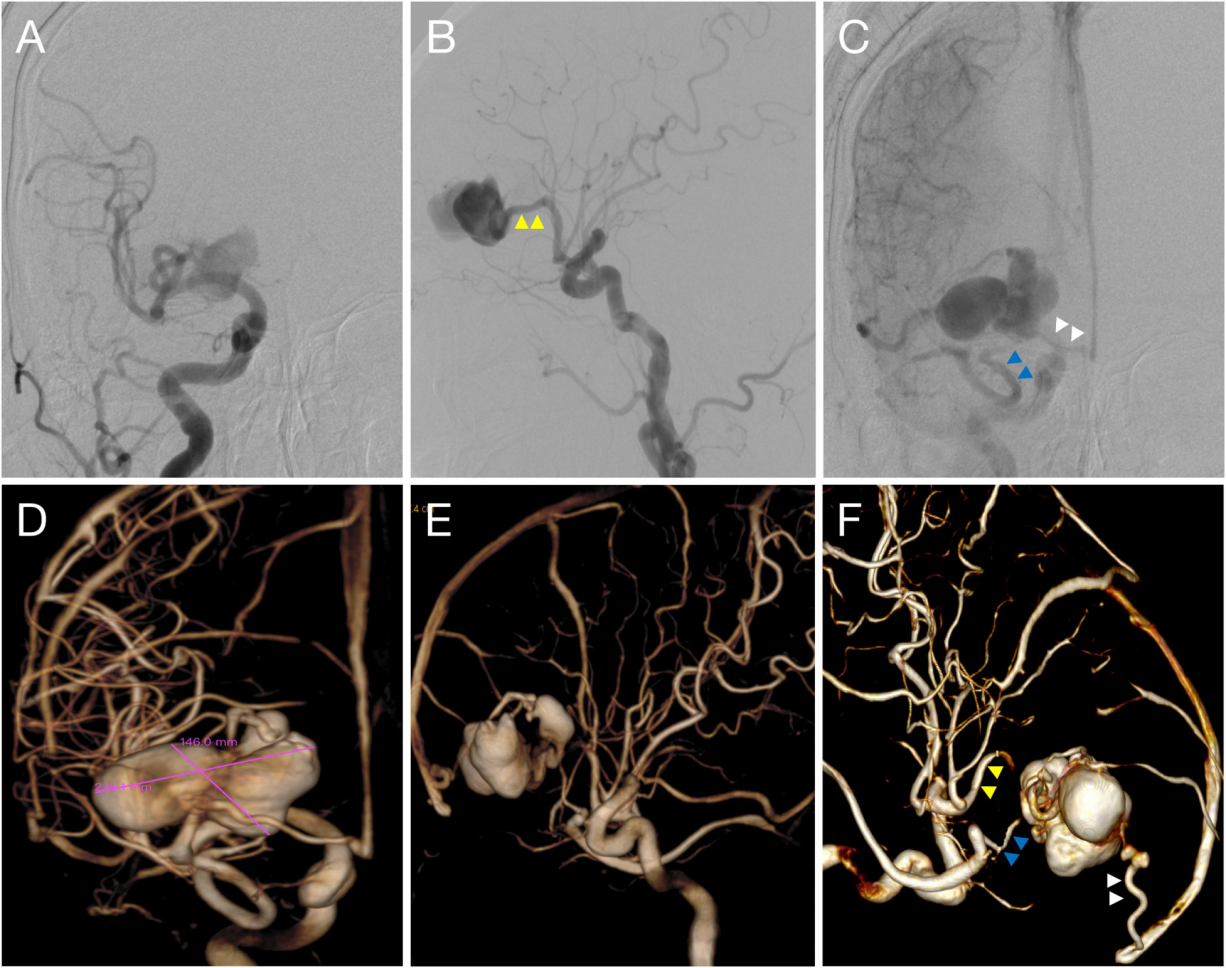
Preoperative right carotid DSA (anteroposterior: **A**; lateral: **B**) demonstrates a large multilobulated venous pouch supplied by a single feeder from the right MCA M2 branch (yellow triangle). The venous phase **(C)** shows predominant drainage to the sphenoparietal sinus (blue triangle) with partial drainage to the superior sagittal sinus (white triangle). 3D-DSA **(D,E,F)** further delineates the giant multilobulated pouch and its dual venous drainage (white and blue triangles).

Subsequent digital subtraction angiography (DSA) confirmed a PAVF. The fistula was located on the frontal cortical surface, supplied by a single arterial feeder arising from the M2 segment of the right MCA (Figures 2A,B,F), with venous drainage into the superior sagittal sinus and the sphenoparietal sinus (Figures 2C–F). A giant multilobulated venous pouch was present (Figures 2D,E). No nidus or dural arteriovenous fistula was identified.

**Figure 3:**
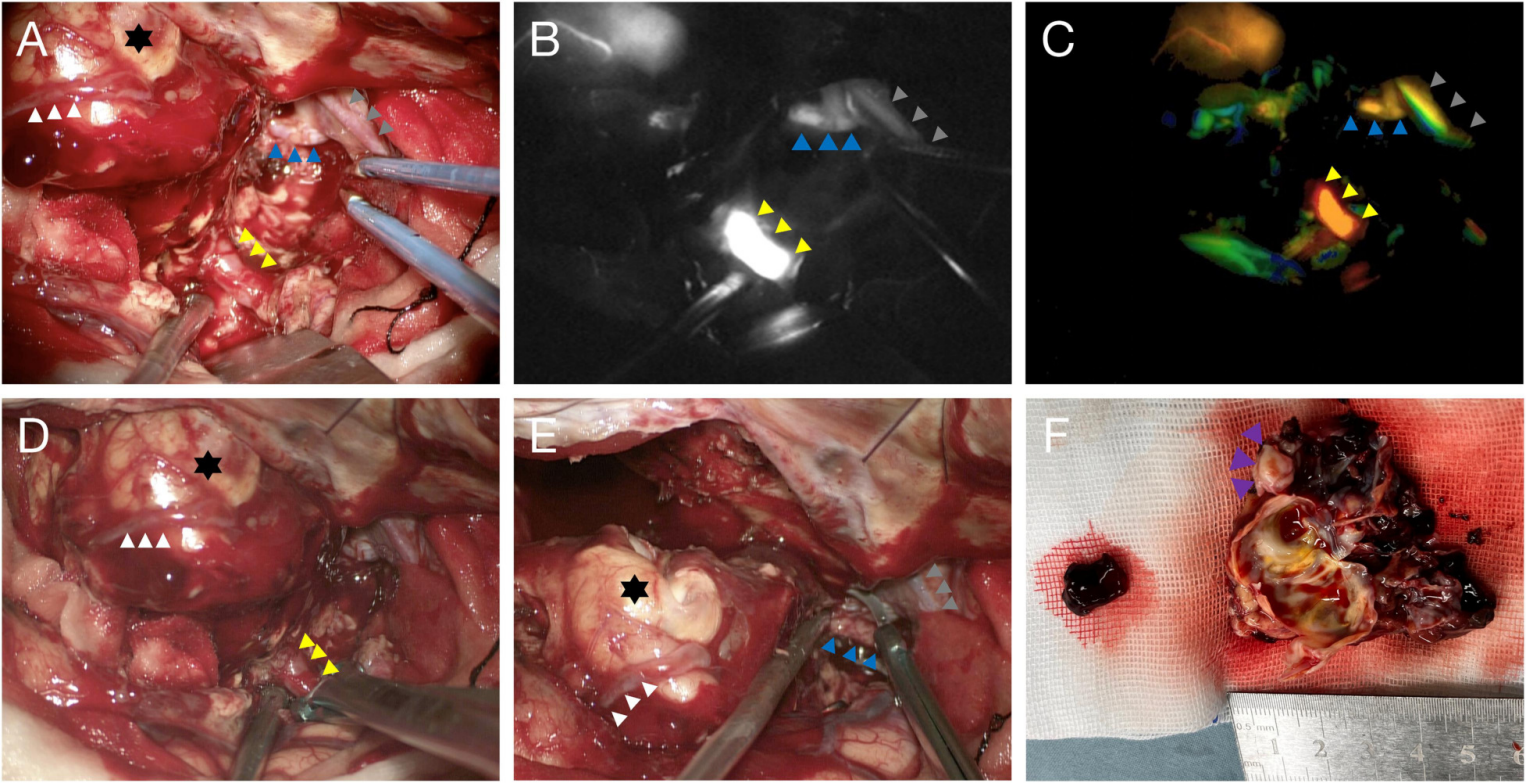
Microsurgical exposure shows the giant venous pouch with the MCA feeder (yellow triangle) and dual draining veins to the sphenoparietal sinus (blue triangle) and superior sagittal sinus (white arrow); the sylvian vein is arterialized (gray triangle) **(A)** intraoperative ICG angiography **(B)** and FLOW800 analysis **(C)** confirm the feeding and draining pathways. After clip occlusion of the feeder, the pouch is dissected along the hematoma cavity **(D)** The venous pouch collapses (black asterisk), and the deep draining vein is then occluded **(E)** Opening of the pouch reveals intraluminal thrombus and focal calcification (purple triangle); maximal pouch diameter is ∼50 mm **(F).**

The next day, the patient underwent right frontal hematoma evacuation and microsurgical excision of the PAVF. Intraoperatively, a giant venous pouch was found on the frontal cortex. A prominent feeder from the M2 segment of the MCA supplied the pouch. Cortical draining veins on the pouch surface coursed to the superior sagittal sinus, while a large deep draining vein extended to the sphenoparietal sinus. The Sylvian draining vein appeared arterialized (Figure 3A). Real-time indocyanine green (ICG) angiography confirmed the feeding artery and draining veins (Figure 3B), and FLOW 800 analysis further demonstrated arterialized flow within the venous pouch and the deep draining vein to the sphenoparietal sinus (Figure 3C). The feeding artery was first occluded with a permanent aneurysm clip, after which the multilobulated venous pouch was progressively dissected around the hematoma and the cortical draining vein to the superior sagittal sinus was divided (Figure 3D). As flow was interrupted, the pouch gradually softened and collapsed, and the Sylvian draining vein regained a normal venous color (Figure 3E). After complete mobilization of the venous pouch, a temporary clip was applied to the large deep draining vein to the sphenoparietal sinus. With no adverse changes observed, the vein was coagulated and divided, and the temporary clip was removed (Figure 3E). The largest diameter of the abnormal pouch was approximately 5 cm, with multiple loculations. Thrombus was observed within the cavities after opening, and focal calcification was present on the pouch surface (Figure 3F).

**Figure 4:**
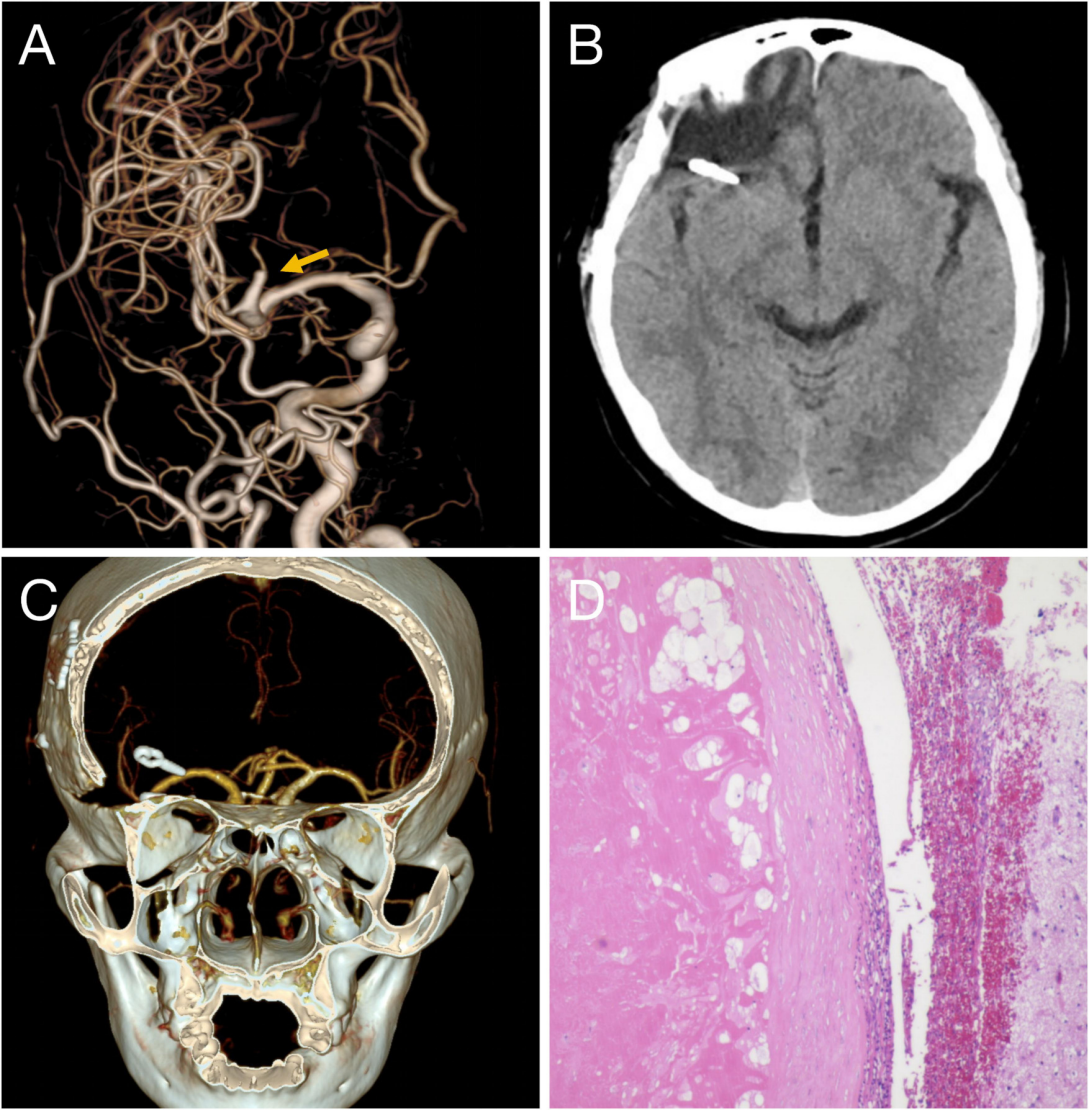
Postoperative DSA confirms complete obliteration of the fistula, with the feeding artery from the right MCA M2 segment completely occluded **(A)**. postoperative CT shows complete evacuation of the right frontal hematoma **(B)**. CTA at 3 months demonstrates no recurrence **(C)**. Histopathology shows a vascular malformation with focal calcification **(D)**.

Postoperatively, the patient was alert and had no neurological deficits. CT on postoperative day 1 confirmed complete hematoma removal without infarction (Figure 4B). Follow-up DSA on postoperative day 4 demonstrated complete interruption of the right MCA M2 feeding branch and total obliteration of the fistula and abnormal draining veins (Figure 4A). Pathology revealed a vascular malformation with focal calcification (Figure 4D). The patient was discharged uneventfully on postoperative day 6. At 3-month follow-up, CTA showed no vascular abnormality (Figure 4C), she remained in good condition with no neurological complaints.

## Literature review methodology

We conducted a targeted narrative review to contextualize this adult intracranial PAVF case. PubMed/MEDLINE was searched from inception to January 1, 2026 using the terms “pial arteriovenous fistula”/“pial AVF” combined with “adult,” and reference lists of relevant articles were screened. We included original adult (≥18 years) intracranial PAVF case reports or case series with extractable data on angioarchitecture, treatment, and outcomes. We excluded dural AVFs, AVMs with a nidus, spinal lesions, pediatric-only studies, and non-original or abstract-only publications.

## Discussion

Pial arteriovenous fistula (PAVF) is an uncommon cerebrovascular lesion defined by a direct high-flow shunt between one or a few pial/cortical arteries and a cortical vein or venous pouch without an intervening nidus. Published data indicate that only several hundred non-galenic PAVFs have been reported to date, pediatric cases predominate, whereas adult cases are mostly isolated reports or small series, underscoring that adult PAVF represents an exceptionally uncommon subgroup (4, 6, 7).

Across the age spectrum, PAVF shows differences in etiology and natural history. In children, especially neonates and infants, the massive shunt flow can cause congestive heart failure, increased intracranial pressure, or macrocephaly, and the condition is generally attributed to congenital cerebrovascular maldevelopment (4, 6). With increasing age, pediatric presentations shift toward intracranial hemorrhage, seizures, and focal deficits (4). In contrast, reported adult PAVFs are frequently associated with acquired factors such as head trauma, venous sinus thrombosis, prior craniotomy, or endovascular procedures. Nonetheless, sporadic cases, such as this patient we presented here, can occur in the absence of an identifiable trigger (7, 8). Clinically, adult PAVF rarely presents with heart failure, instead, hemorrhage, headache, seizures, or focal neurological deficits predominate (1, 9). Our 56-year-old patient presented with exercise-related sudden severe headache and right frontal intracerebral hemorrhage, consistent with the typical adult pattern and indicating that adult PAVFs still carry substantial hemorrhagic potential.

Venous-side pathology plays a critical role in the natural course of PAVF. Venous varices/giant venous pouches are common diagnostic clues and major sources of hemorrhage and mass effect (8). Large pediatric cohorts show that venous outflow obstruction, sinus thrombosis, and ventriculomegaly correlate with poor outcome, and the annual hemorrhage risk without treatment is estimated at 3%–4% (10). In our adult patient, DSA revealed the presence of at least two interconnected venous pouches. Intraoperative exploration confirmed the rupture of one such pouch as the source of hemorrhage, underscoring that varix/pouch rupture constitutes a crucial, and potentially novel, hemorrhagic mechanism in adult PAVFs.

Given the aggressive natural history and poor prognosis with conservative management, current consensus supports definitive treatment aimed at complete fistula occlusion whenever the patient can tolerate intervention (8). Unlike AVM management, which may require extensive parenchymal or nidus resection, the therapeutic goal for this condition is to achieve selective “flow disconnection” of the single high-flow shunt. This approach aims to preserve normal venous drainage while effectively relieving mass effect (11). Pediatric PAVFs often require early endovascular embolization to rapidly reduce shunt flow and cardiovascular burden (4). Adult PAVFs usually lack heart-failure emergencies, some hemodynamically stable, mildly symptomatic elderly patients may be candidates for close observation, and adult management therefore emphasizes balancing hemorrhage prevention against overtreatment (4, 9).

Both microsurgical and endovascular treatments can achieve high occlusion rates. Systematic reviews suggest similar angiographic cure across surgical, endovascular, and combined strategies (approximately 86%–89%) (3, 12). Endovascular embolization is often preferred for deep, infratentorial, or skull base PAVFs, or for lesions with multiple feeders (8). However, high-flow single-channel shunts can be challenging. Catheterization may be difficult in small or tortuous feeders. Reflux or distal migration of embolic material can occur. Non-target embolization may cause infarction or hemorrhage. Abrupt changes in venous outflow may also trigger venous thrombosis, edema, or hydrocephalus (8). Microsurgery is generally favored for superficial hemispheric PAVFs, especially when there is an intracerebral hematoma or a large venous pouch with mass effect (13, 14). It allows direct fistula disconnection while preserving normal veins. It also enables hematoma evacuation and, when feasible, pouch resection in the same session. Cure can be confirmed immediately with intraoperative adjuncts. In this patient, several lesion-specific factors favored surgery: (i) the fistula was superficial and supratentorial, making direct exposure feasible; (ii) the patient presented with a large intracerebral hematoma, for which craniotomy allowed simultaneous hematoma evacuation and immediate decompression; (iii) the venous pouch was giant and multilobulated, raising concerns that embolization might leave a thrombosis-prone residual pouch or precipitate abrupt venous outflow changes; and (iv) the feeder originated from an MCA M2 branch, where embolic reflux or non-target embolization could risk ischemia in eloquent territories. In addition, high-flow shunts increase the risk of distal migration of liquid embolic agents and incomplete penetration of the fistulous point, potentially resulting in residual shunting and rebleeding. Therefore, a direct microsurgical strategy aimed at definitive “flow disconnection” and pouch management was considered the most reliable approach in this specific anatomical and clinical context.

Intraoperative indocyanine green videoangiography (ICG-VA), especially when paired with FLOW800 analysis, has been increasingly used to provide real-time, semi-quantitative information on vascular flow dynamics. Prior work in AVM/dAVF surgery suggests that FLOW800 can help identify early arterialized veins, delineate patient-specific venous drainage, and guide decisions regarding venous sacrifice, potentially reducing complications (15). In our case, ICG-VA and FLOW800 confirmed the feeding artery and draining veins before clip application, and helped verify venous de-arterialization after feeder occlusion, supporting a safe sequence of dissection and pouch management. This is particularly relevant in adult PAVF with dual venous drainage, where premature interruption of a functional outflow route could risk venous infarction.

Pathology demonstrated a vascular malformation with focal calcification, and intraoperative inspection revealed thrombus within the pouch cavities. These findings are consistent with chronic high flow venopathy in a longstanding venous pouch/varix, where repeated wall stress may promote mural injury, thrombosis with organization, and dystrophic calcification. Similar “thrombosed/calcified varix” phenomena secondary to pial single-channel fistulas have been described in the literature and support the concept that venous pouch pathology can evolve over time and may contribute to fragility or hemorrhagic presentation (16). From a surgical standpoint, once the shunt is definitively disconnected, resection of the diseased pouch can remove a potentially unstable or mass-effect–producing structure.

Although adult PAVF is rare, this case has several features that distinguish it from most previously reported adult PAVFs. First, the lesion was associated with an unusually large (approximately 5 cm), multilobulated venous pouch, which acted not only as the draining structure but also as a space-occupying component in the setting of intracerebral hemorrhage. Second, angiography demonstrated dual venous drainage to both the superior sagittal sinus and the sphenoparietal sinus, indicating more complex venous outflow than the single-drainage pattern commonly described in adult cases; this feature also increased the importance of preserving functional venous pathways during treatment. Third, our management highlights the specific utility of intraoperative ICG video angiography with FLOW800 analysis in adult PAVF surgery: it facilitated real-time identification of the fistulous point and arterialized drainage, supported a stepwise strategy for venous management after feeder occlusion, and provided immediate confirmation of flow reduction and shunt disconnection. Together, these anatomic characteristics and the FLOW800-guided microsurgical workflow represent the main unique contribution of this report to the limited adult PAVF literature.

## Conclusion

Adult PAVF is exceptionally rare, yet the high-flow shunt and associated venous varix/pouch confer a substantial risk of hemorrhage. For superficial supratentorial adult PAVFs presenting with hematoma and mass effect, microsurgical hematoma evacuation combined with fistula disconnection and venous pouch management can be safe and curative.

## Data Availability

The original contributions presented in the study are included in the article/Supplementary Material, further inquiries can be directed to the corresponding author.
